# The Effect of Precipitate Evolution on Austenite Grain Growth in RAFM Steel

**DOI:** 10.3390/ma10091017

**Published:** 2017-09-01

**Authors:** Biyu Yan, Yongchang Liu, Zejun Wang, Chenxi Liu, Yonghong Si, Huijun Li, Jianxing Yu

**Affiliations:** 1 State Key Lab of Hydraulic Engineering Simulation and Safety, School of Materials Science & Engineering, Tianjin University, Tianjin 300350, China; 13212273263@163.com (B.Y.); licmtju@163.com (Y.L.); huijun@uow.edu.au (H.L.); wayxs1122@163.com (J.Y.); 2 Collaborative Innovation Center for Advanced Ship and Deep-Sea Exploration, Shanghai Jiao Tong University, Shanghai 200240, China; 3 Tianjin Special Equipment Inspection Institute, Tianjin 300192, China; wangzejun814@sina.com (Z.W.); 13920619513@163.com (Y.S.)

**Keywords:** RAFM steel, austenite, precipitate, dissolution, coarsening

## Abstract

To study the effects of various types of precipitates and precipitate evolution behavior on austenite (size and phase fraction) in reduced activation ferritic/martensitic (RAFM) steel, RAFM steel was heated to various austenitizing temperatures. The microstructures of specimens were observed using optical microscopy (OM) and transmission electron microscopy (TEM). The results indicate that the M_23_C_6_ and MX precipitates gradually coarsen and dissolve into the matrix as the austenitizing temperatures increase. The M_23_C_6_ precipitates dissolve completely at 1100 °C, while the MX precipitates dissolve completely at 1200 °C. The evolution of two types of precipitate has a significant effect on the size of austenite. Based on the Zener pinning model, the effect of precipitate evolution on austenite grain size is quantified. It was found that the coarsening and dissolution of M_23_C_6_ and MX precipitates leads to a decrease in pinning pressure on grain boundaries, facilitating the rapid growth of austenite grains. The austenite phase fraction is also affected by the coarsening and dissolution of precipitates.

## 1. Introduction

Reduced activation ferritic/martensitic (RAFM) steel has been considered as a promising candidate material for the first wall and blanket structures of demonstration (DEMO) and commercial fusion reactors, due to its low thermal expansion coefficient, high thermal conductivity, and favorable radiation swelling resistance [1,2]. Considerable attention has been paid to the research and development of RAFM steels in Japan, Europe, and the US [3,4]. However, it is still necessary to improve the mechanical properties of RAFM steels (i.e., strength and toughness) [5]. 

In general, austenite has an important effect on the mechanical properties of steels after cooling [6]. The coarsening of austenite grain would debase the mechanical properties. To refine the grain size of RAFM steels, alloying elements such as chromium (Cr), vanadium (V), and tantalum (Ta) are generally added [7,8]. These alloying elements can contribute to the formation of second phase particles (M_23_C_6_ and MX), both of which can affect the austenite grain size at high temperatures [9,10]. The finely dispersed precipitates can lower the grain growth rate and retard the austenite grain growth by the pinning effect (pinning pressure) on the austenite grain boundary [11,12]. Rath et al. [13] proposed that the normal austenite grain growth in an isothermal heat treatment was driven (driving pressure) by the reduction of the total surface energy. Meanwhile, the particles existed in the matrix would inhibit the migration of the grain boundary by the pinning pressure [14]. For decades, considerable research work has been aimed at investigating the retardation of austenite grain growth by the second phase particles. Some results have shown that the second phase particles inhibit the austenite grain growth by retarding the migration of the grain boundary [15,16]. The driving pressure for the normal austenite grain growth would be decreased due to the pinning pressure. Moreover, during heat treatment, the coarsening and dissolution of second phase particles would occur, leading to changes in the size and volume fraction of precipitates. This would in turn result in a change in pinning pressure, affecting the austenite grain size [17]. Besides, the dissolution of precipitates in the matrix also causes the change of the austenite phase fraction, which could affect the mechanical properties of materials [18]. However, studies on the effect of the different types of precipitates and precipitation-dissolution behaviors on austenite (size and fraction) in RAFM steels are seldom reported, and require more attention.

In this work, a study on the austenite growth behavior of RAFM steel was carried out. Two types of precipitates (M_23_C_6_ and MX) as well as the relationship between precipitation evolution (coarsening and dissolution) behavior and austenite (size and phase fraction) in RAMF steels were investigated in detail. 

## 2. Experimental Procedure

**Figure 1:**
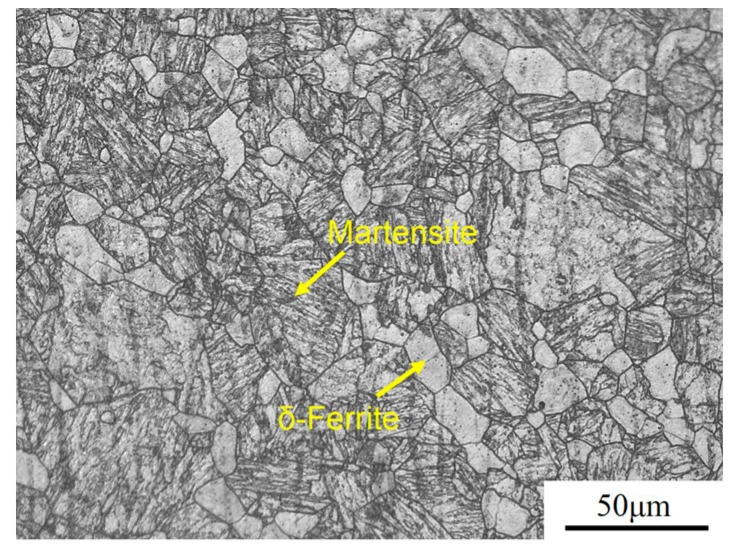
Optical micrographs showing the microstructure of the original reduced activation ferritic/martensitic (RAFM) steel after casting and hot processing.

The experimental steel investigated in this research is a 9% Cr RAFM steel, and its chemical composition is given in Table 1. Figure 1 displays the optical micrograph of the RAFM steel prior to the normalizing treatment. The initial material was machined from an ingot subjected to casting and forging processing. After casting and forging processing, the microstructure of the RAFM steel consisted of martensite and small amount of δ-ferrite. The δ-ferrite may have originated from the casting treatment, and the fraction of the δ-ferrite phase fraction is about 18%.

To obtain M_23_C_6_ and MX precipitates, the initial material of the RAFM steel was normalized at 1050 °C for 0.5 h and then tempered at 750 °C for 1.5 h. Cylindrical specimens with a length of 10 mm and a diameter of 4.5 mm were machined from the tempered initial material. The cylindrical specimens were heated (at a rate of 200 °C min^−1^) to different austenitizing temperatures (900, 1000, 1100, and 1200 °C) for 400 s, followed by water quenching. To reveal the prior austenite grain boundaries, the mounted samples were polished and etched in a mixed solution of water (100 mL), picric acid (2 g), and detergent (2 mL) at 70 °C for 3 min. The microstructure was characterized by optical microscopy (OM, Leica DMI 8, Leica, Solms, Germany) and transmission electron microscopy (TEM, JEM-2100f, JEOL, Akishima, Tokyo, Japan). Image analyzing software (Image Pro Plus 6.0, Media Cybernetics, MD, America) and the linear intercept method were adopted to determine the size and phase fraction of prior austenite grains. The morphology, size, and distribution of second phase particles were examined by the carbon extraction replica technique. Finally, the Vickers hardness was determined with the MH-6 Vickers hardness tester using a 50-N load for 5 s.

To accurately analyze the change of second phase particles, the volume fraction of precipitates was calculated by [19]:

(1) $$f=\frac{N\frac{4\pi }{3}r^{3}}{SD}$$

where f is the volume fraction of precipitates, *N* is the number of precipitates per area, r is the radius of precipitates, *S* is the specific area for estimation, and *D* is the equivalent diameter of precipitates. The MX precipitates in RAFM steels are mainly spherical, and thus Equation (1) is suitable to estimate the volume fraction of MX precipitates. However, most M_23_C_6_ precipitates exhibit rectangular morphology. Therefore, the edge sizes of the rectangles must be converted to a hypothetical sphere of radius *r* by the following equation [20]:

(2) $$r=\sqrt{\frac{L_{A}L_{B}}{\pi }}$$

where $L_{A}$ and $L_{B}$ are the measured edge sizes of the M_23_C_6_ precipitates.

## 3. Results

**Figure 2:**
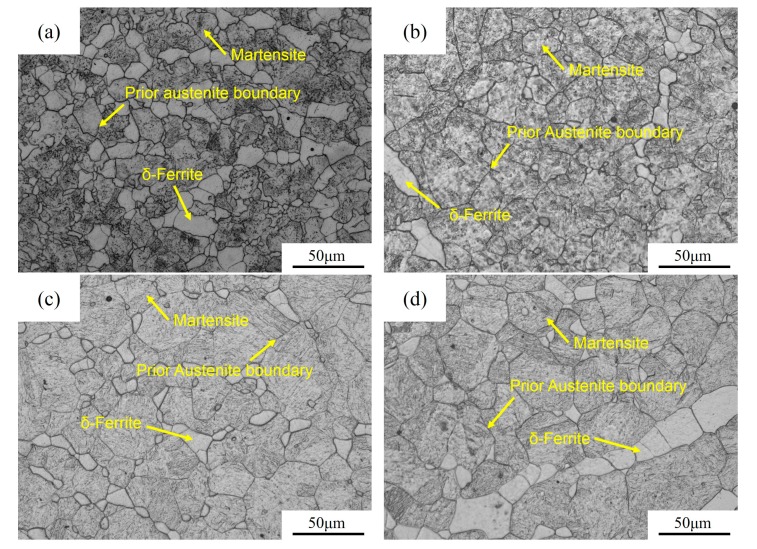
Optical micrographs showing the prior austenite grain boundaries, holding at: (**a**) 900 °C; (**b**) 1000 °C; (**c**) 1100 °C; and (**d**) 1200 °C for 400 s.

The optical micrographs of the specimens austenitized at various temperatures are shown in Figure 2. The prior austenite boundary can be readily seen. With the increase of the austenitizing temperature, the prior austenite grains tend to gradually coarsen.

**Figure 3:**
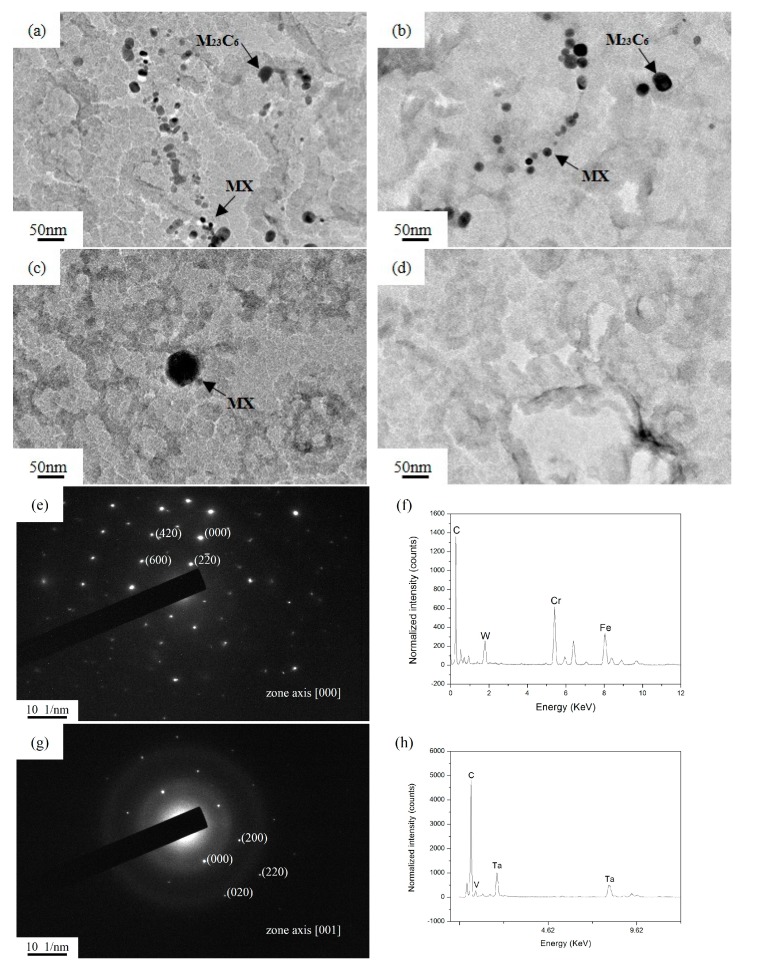
TEM micrograph of extraction replica, holding at: (**a**) 900 °C; (**b**) 1000 °C; (**c**) 1100 °C; and (**d**) 1200 °C for 400 s; (**e**–**h**): The selected area electron diffraction (SAED) pattern and the energy dispersive spectroscopy (EDS) analysis of M_23_C_6_ and MX precipitates, respectively.

Figure 3 represents the particles present in the samples held at different austenitizing temperatures. The distribution of the precipitates in a line suggests that the majority of them are distributed on the prior austenite boundary. The selected area electron diffraction (SAED) pattern and the energy dispersive spectroscopy (EDS) analysis of the rectangular precipitates are shown in Figure 3e–f. Based on the EDS analysis, the rectangular precipitates are rich in Cr. Combined with the diffraction pattern, the precipitates are identified as M_23_C_6_. The circular precipitates are identified as MX containing Ta and V.

**Figure 4:**
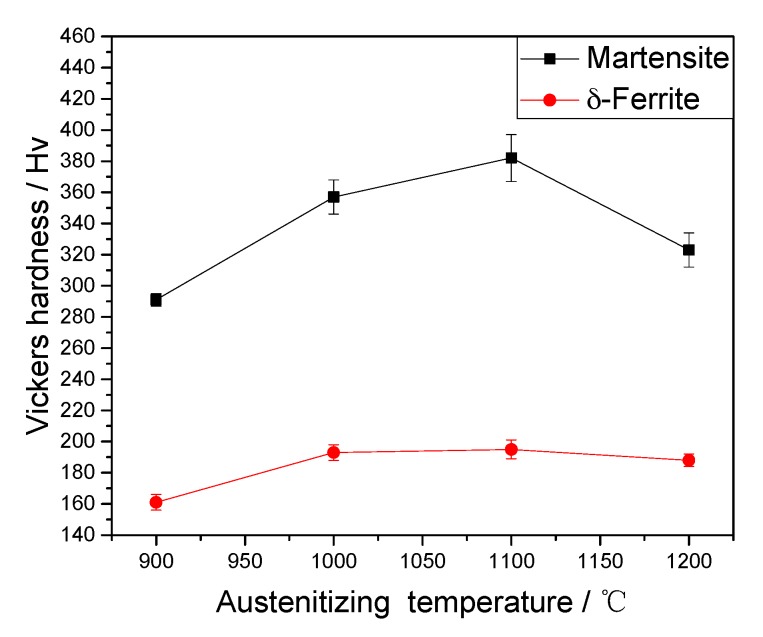
Effect of the austenitizing temperature on the Vickers hardness of δ-ferrite and martensite.

Since the microstructure of RAFM steel consists of martensite and δ-ferrite, the hardness of δ-ferrite and martensite was respectively evaluated, as shown in Figure 4. With the increase of the austenitizing temperature, the hardness of δ-ferrite is nearly constant, while the hardness of martensite first increases and then decreases.

## 4. Discussion

### 4.1. The Evolution of M_23_C_6_ and MX Precipitates 

**Figure 5:**
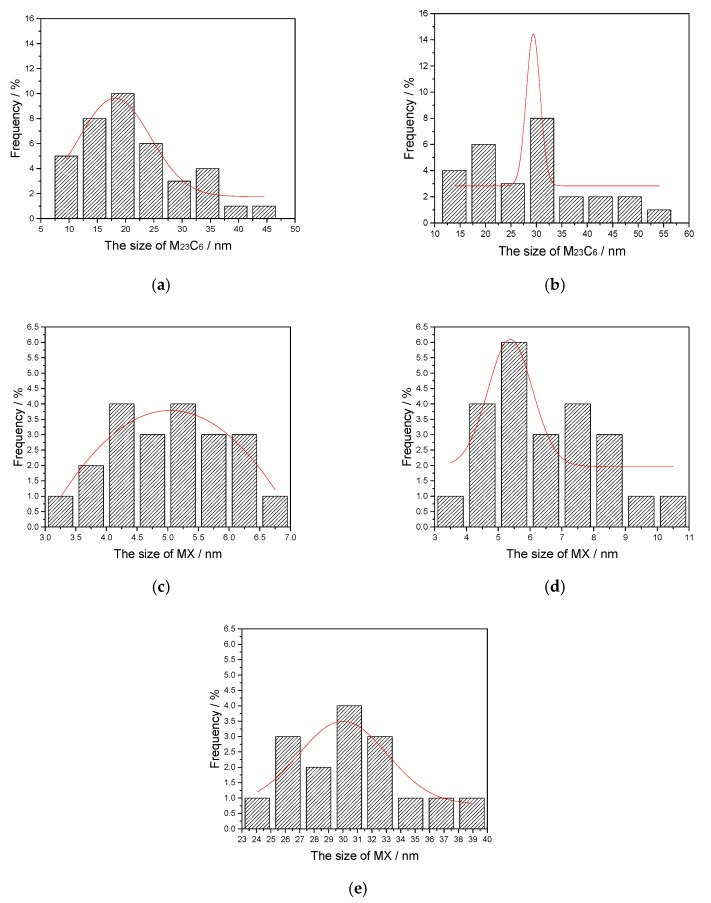
(**a**,**b**) The normal distribution of M_23_C_6_ precipitates size, holding at 900 and 1000 °C for 400 s; (**c**–**e**) The normal distribution of MX precipitates size, holding at 900, 1000, and 1100 °C for 400 s. The red curve represents the normal distribution curve of carbide size.

Volume fraction and size (a hypothetical sphere of radius) of the precipitates were evaluated by Equations (1) and (2), and the results are shown in Table 2. As displayed in Figure 5, the size (a hypothetical sphere of radius) distribution of particles follows atypical normal distribution (Gaussian distribution). With the increase of the austenitizing temperature, the peak value of precipitates size distribution gradually shifts to the right. This indicated that precipitates would coarsen with the raise of the austenitizing temperature.

In Figure 3a–d, the M_23_C_6_ precipitates coarsen as the austenitizing temperature increases. Meanwhile, the stability of M_23_C_6_ precipitates decreases gradually, and some M_23_C_6_ precipitates with a small size will dissolve. The decrease in the volume fraction of M_23_C_6_ precipitates demonstrates this point (Table 2). When the austenitizing temperatures are higher than 1000 °C, the M_23_C_6_ precipitates completely dissolved (Figure 3c). On the other hand, compared with M_23_C_6_ precipitates, the average size and volume fraction of MX precipitates also respectively increase and decrease when the austenitizing temperature increases. However, the changes in size and volume fraction of MX precipitates were relatively smaller than those of M_23_C_6_ precipitates at 900–1000 °C. According to previous research [21], MX precipitates have a higher thermal stability against coarsening and dissolution when the austenitizing temperature is below 1100 °C. Thus, MX precipitates show slower coarsening and dissolution rates at 900–1000 °C. With the increase of the austenitizing temperature, the coarsening of MX precipitates is evident at 1100 °C (from 5.56 to 29.35 nm) and a sharp reduction in its amount can also be noted (Figure 3c). The MX precipitates finally dissolve at 1200 °C.

### 4.2. Effects of Precipitates on Austenite Sizes

**Figure 6:**
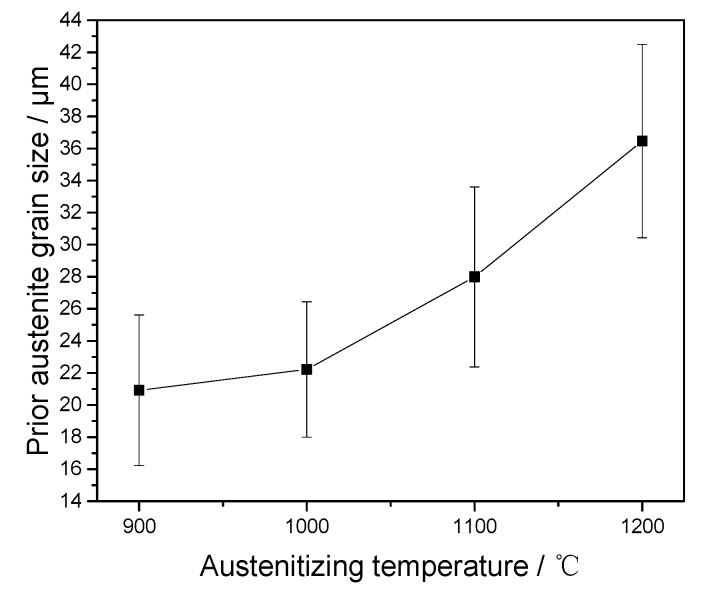
Measured prior grain sizes of austenite of the samples upon different austenitizing temperatures.

Previous studies [10,11,12,13,14] usually only considered the effect of one type of precipitate on austenite growth, while in this work, two types of carbides with different sizes and thermal stabilities are considered. The austenite grain sizes in this study were determined with the linear intercept method. Figure 6 displays the average sizes of prior austenite grains at each austenitizing temperature. As the austenitizing temperature increases from 900 to 1200 °C, the austenite grain sizes are respectively determined as 20.92, 22.22, 27.99, and 36.46 μm. The average sizes of prior austenite grains under the different austenitizing temperatures increase as the austenitizing temperature increases, which can be explained as the prior austenite boundary mobility increasing as the austenitizing temperature increases.

Generally, the sizes of the prior austenite grains in the steels regularly increase with the increase of the austenitizing temperature [22,23,24]. Nevertheless, between the austenitizing temperatures of 900 and 1000 °C, the growth of prior austenite grain in this study is not sensitive to austenitizing temperature and is significantly retarded. The prior austenite grain size increases obviously when the austenitizing temperatures are higher than 1000 °C. The austenite grains at lower austenitizing temperatures exhibit a slower growth rate than those at higher austenitizing temperatures. Zener et al. proposed that the precipitates on austenite grain boundaries would impede the austenite grain growth by a pinning pressure [25]. This may account for the confined growth of austenite grains in this work. It implies the retardation of grain growth due to precipitates.

The microstructure evolution of randomly distributed precipitates (i.e., dissolution or coarsening) during heat treatment has a significant effect on austenite grain growth. To evaluate this effect, Zener proposed the following equation [26]:

(3) $$P_{z}=\beta \frac{\gamma \cdot f}{r}$$

where f is the volume fraction of pinning precipitates, r is average radius of precipitates, β is a dimensionless constant (β=12) [27], and γ is the interfacial energy. 

When the carbon content *C* is below 0.8% in wt %, *γ* can be calculated by [28]:

(4) $$\gamma =\left(0.8-0.35C^{0.68}\right)$$

The interfacial energy calculated in this study is 0.76 $J\cdot m^{-2}$. In the presence of multiple precipitates distributions, the final pinning pressure $P_{p}$ can be calculated by [25]:

(5) $$P_{p}=\beta \cdot \sum^{\text{​}}\frac{\gamma \cdot f_{i}}{r_{i}}$$

where the summation index *i* represents all precipitate families, i.e., in this study, M_23_C_6_ and MX precipitates.

**Figure 7:**
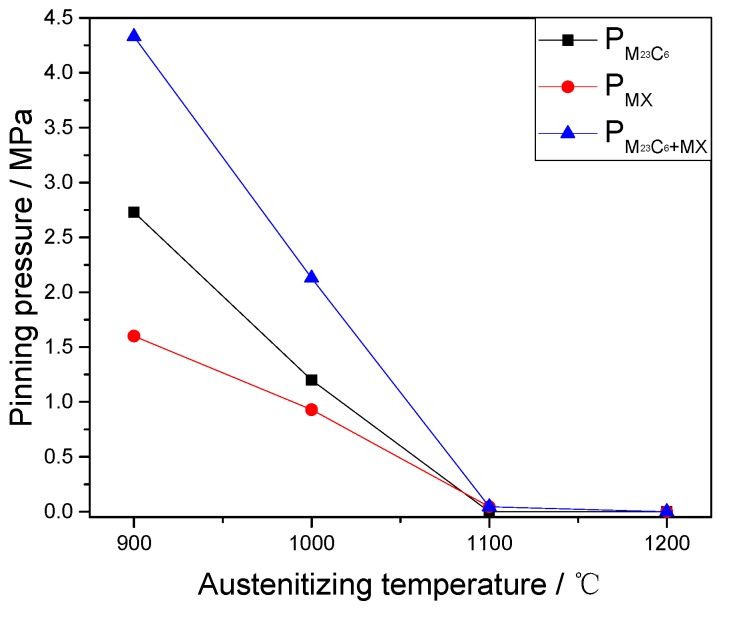
The pinning pressure at different austenitizing temperatures.

Replacing the average radius and volume fraction of the M_23_C_6_ and MX precipitates into Equations (3) and (5), the pinning pressure ($P_{z}$ and $P_{p}$) on the grain boundaries at different austenitizing temperatures can be obtained, as shown in Figure 7. The pinning pressure decreases with the increase of the austenitizing temperature. This is caused by the reduction in the number of pinning positions resulting from dissolution of precipitates. According to Equation (3), the increase of precipitates size due to coarsening is also an important factor. In addition, from 900 to 1000 °C, the reduction of the pinning pressure ($∆P_{M23C6}=2.73$ MPa) caused by the M_23_C_6_ precipitates is more than that ($∆P_{\mathrm{MX}}=1.6$ MPa) caused by the MX precipitates, due to the lower thermal stability of M_23_C_6_ precipitates. When the austenitizing temperature is in the range of 1000–1100 °C, the entire dissolution of M_23_C_6_ precipitates results in the disappearance of the pinning pressure $P_{M23C6}$. Meanwhile, the serious coarsening and dissolution of MX precipitates lead to a significant decrease of the pinning pressure $P_{\mathrm{MX}}$, from 0.93 to 0.045 MPa. Then, the complete dissolution of MX precipitates also causes the decrease of the pinning pressure $P_{\mathrm{MX}}$ at 1200 °C.

Overall, the change of pinning pressures $P_{M23C6}$ and $P_{\mathrm{MX}}$ causes the reduction and removal of the final pinning pressure $P_{M23C6+\mathrm{MX}}$, because of the coarsening and dissolution of M_23_C_6_ and MX precipitates. The contributions of the pinning effect from different types of carbides are dependent on their thermal stabilities. Figure 5 shows that the prior austenite grain growth rate gradually increases as the austenitizing temperature increases, especially at temperatures over 1000 °C. This suggests that the microstructure evolution of two types of precipitate has a close relationship with the retarded growth of the austenite grain. The variety of precipitates reduces the pinning effect on the grain boundary, which is beneficial to the austenite growth. 

### 4.3. Effect of Precipitates on Austenite Phase Fraction

**Figure 8:**
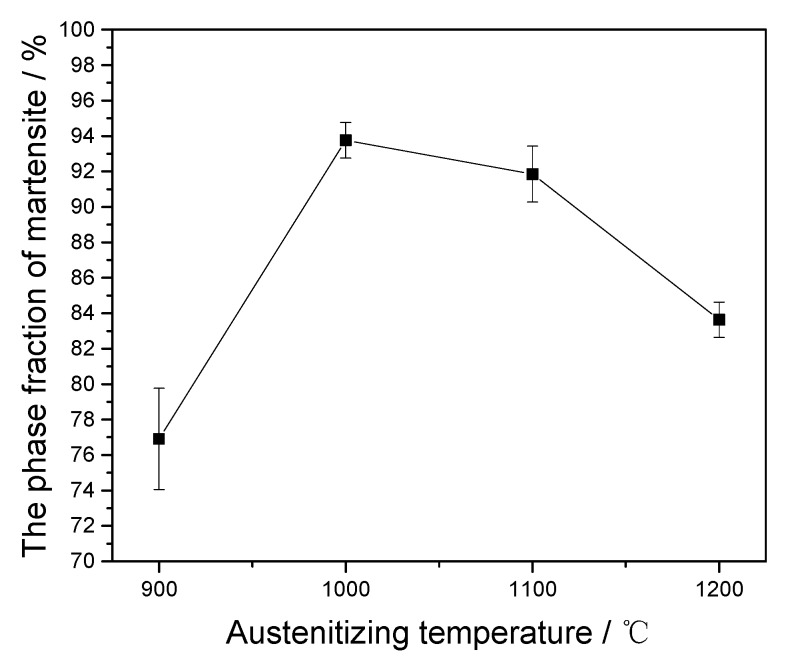
The area fraction of martensite of the samples upon different austenitizing temperatures.

The average phase fraction of martensite under different austenitizing temperatures is illustrated in Figure 8. In this work, the martensite phase fractions in the final microstructures are considered as the austenite phase fractions at high temperatures. The average phase fraction of austenite increases first and then decreases as the austenitizing temperature increases. When the austenitizing temperature is 900 °C, the austenite phase fraction is only 76.91%. As shown in the Figure 1, some amount of δ-ferrite already exists in the original microstructure of the RFAM steel in this study. On the other hand, the austenitizing temperature of 900 °C is just at the *α/γ* two-phase region [29]. This indicates that the austenitizing of RAFM steel is incomplete at 900 °C. Some amount of α-ferrite may remain after cooling. As shown in Figure 4, the Vickers hardness of δ-ferrite at 900 °C is smaller than that at 1000–1200 °C, which also indicates the existence of α-ferrite. In addition, the short holding time (400 s) is also the reason why the austenite phase fraction is only 76.91%. However, when the austenitizing temperature is 1000 °C, the phase fraction of austenite is 93.76%, which is much larger than the phase fraction of 76.91% at the austenitizing temperature of 900 °C. As the austenitizing temperature increases from 900 to 1000 °C, some small precipitates begin to gradually dissolve. This is beneficial to the nucleation and growth of austenite. The pinning pressure caused by the M_23_C_6_ precipitates gradually decreases due to the dissolution of carbides at 900–1000 °C. This can promote the increase of austenite phase fraction. While the austenitizing temperature increases from 1000 to 1200 °C, the phase fraction of austenite gradually decreases, owing to the formation of δ-ferrite at high temperatures. Thus, the phase fraction of austenite is affected by the carbide dissolution and the δ-ferrite/austenite phase transformation. 

### 4.4. Vickers Hardness

The growth of austenite grains and the dissolution of precipitates would affect the Vickers hardness of martensite. When the austenitizing temperature increases from 900 to 1100 °C, both the carbide dissolution and austenite growth occur. The precipitate dissolution would increase the amount of carbon saturated in martensite, resulting in the increase in hardness of martensite (291 to 382 Hv). The elements dissolved from precipitates, such as Mo, V, and Cr, would be beneficial to solution strengthening [30]. However, the increase of prior austenite grain size would lead to the increase of the block size in lath martensite [31,32]. The increase of block size would be disadvantageous to sub-boundary hardening, which the block boundaries result in Reference [33]. In addition, the hardness of martensite is essentially caused by the dislocation motion in martensite. The movement of dislocation motion is hindered by the block boundary. The larger block size implies that the number of block boundaries would decrease, which also is disadvantageous to the increase in hardness of martensite [34,35]. When the austenitizing temperature increases from 900 to 1100 °C, the precipitate dissolution on the hardness of martensite is dominated, thus the hardness of martensite is increased. While the temperature reaches 1200 °C, the effect of austenite growth on the hardness of martensite is predominant, and the hardness decreases. Besides, the formation of a considerable amount of δ-ferrite would also consume the solution strengthening elements, thus decreasing the hardness of martensite.

## 5. Summary and Conclusions

In the present work, the effects of different types of precipitates and precipitate evolution behaviors on austenite in RAMF steels were investigated. The conclusions can be summarized as follows:

(1) The M_23_C_6_ and MX precipitates gradually coarsen and dissolve into the matrix as the austenitizing temperature increases. The M_23_C_6_ precipitates dissolve completely at 1100 °C, while the MX precipitates dissolve completely at 1200 °C.
(2) The increase of austenite grain size is retarded due to the retarding of two different types of precipitate (M_23_C_6_ and MX) with the sizes of 9–70 nm by exerting a pinning pressure on the grain boundaries. The coarsening and dissolution of M_23_C_6_ and MX precipitates also result in the reduction and removal of the pinning pressure on grain boundaries, which contributes to the free growth of the austenite grains.
(3) The austenite phase fraction increases first and then gradually decreases as the austenitizing temperature increases. The dissolution of the two types of precipitate has an important effect on the change of the austenite phase fraction.
(4) With the increase of austenitizing temperatures, the hardness of δ-ferrite is nearly constant, while the hardness of martensite first increases and then decreases. The precipitate dissolution is propitious to the increase in the hardness of martensite, while the formation of δ-ferrite and prior austenite grain growth is disadvantageous to the increase in the hardness of martensite.

## Figures and Tables

**Table 1:** Chemical compositions of experimental steel (wt %).

C	Cr	W	Mn	Si	V	Ta	Fe
0.04	8.93	1.71	0.44	0.04	0.22	0.073	Bal

**Table 2:** Average volume fraction $f_{a}$ and radius $r_{a}$ of precipitates at different austentitizing temperatures.

Parameter	Precipitate	Austenitizing Temperature/°C			
		900	1000	1100	1200
$f_{a}$	M_23_C_6_	0.00650	0.00380	—	—
	MX	0.00089	0.00066	0.00015	—
$r_{a}$/nm	M_23_C_6_	21.71	28.84	—	—
	MX	5.07	6.50	30.49	—
